# Low immunoglobulin levels affect the course of COPD in hospitalized patients

**DOI:** 10.1186/s13223-023-00762-x

**Published:** 2023-01-29

**Authors:** Nami Shrestha Palikhe, Malcena Niven, Desi Fuhr, Tristan Sinnatamby, Brian H. Rowe, Mohit Bhutani, Michael K. Stickland, Harissios Vliagoftis

**Affiliations:** 1 Division of Pulmonary Medicine, Department of Medicine, Faculty of Medicine and Dentistry, 550 A HMRC, University of Alberta, Edmonton, AB T6G 2S2 Canada; 2 Alberta Respiratory Centre, University of Alberta, Edmonton, AB Canada; 3 Department of Emergency Medicine and Faculty of Medicine and Dentistry, University of Alberta, Edmonton, AB Canada; 4 School of Public Health, University of Alberta, Edmonton, AB Canada; 5 G.F. MacDonald Centre for Lung Health, Covenant Health, Edmonton, AB Canada

**Keywords:** Hypogammaglobulinemia, COPD, Immunoglobulins, AECOPD

## Abstract

**Background:**

Chronic obstructive pulmonary disease (COPD) affects up to 10% of Canadians. Patients with COPD may present with secondary humoral immunodeficiency as a result of chronic disease, poor nutrition or frequent courses of oral corticosteroids; decreased humoral immunity may predispose these patients to mucosal infections. We hypothesized that decreased serum immunoglobulin (Ig) levels was associated with the severity of an acute COPD exacerbations (AECOPD).

**Methods:**

A prospective study to examine cardiovascular risks in patients hospitalized for AECOPD, recruited patients on the day of hospital admission and collected data on length of hospital stay at index admission, subsequent emergency department visits and hospital readmissions. Immunoglobulin levels were measured in serum collected prospectively at recruitment.

**Results:**

Among the 51 patients recruited during an admission for AECOPD, 14 (27.5%) had low IgG, 1 (2.0%) low IgA and 16 (31.4%) low IgM; in total, 24 (47.1%) had at least one immunoglobulin below the normal range. Patients with low IgM had longer hospital stay during the index admission compared to patients with normal IgM levels (6.0 vs. 3.0 days, *p* = 0.003), but no difference in other clinical outcomes. In the whole cohort, there was a negative correlation between serum IgM levels and length of hospital stay (R = − 0.317, p = 0.024). There was no difference in clinical outcomes between subjects with normal and low IgG levels.

**Conclusion:**

In patients presenting with AECOPD, low IgM is associated with longer hospital stay and may indicate a patient phenotype that would benefit from efforts to prevent respiratory infections.

*Trial registration statement*: Retrospectively registered.

## Background

Chronic obstructive pulmonary disease (COPD) is a chronic respiratory disease characterized by airflow obstruction that is not fully reversible [1]. COPD is the third leading cause of death worldwide, affecting around 10% of the world’s population [1] and carries a very high cost for the health care system. Inhalation of tobacco smoke is the major risk factor for development of COPD; however, other factors also contribute to the development of the disease [2, 3]. In addition to airway inflammation, COPD is also characterized by systemic inflammation as shown by an increase in systemic inflammatory markers in these patients [4]. This systemic inflammation is linked to comorbidities such as atherosclerosis and cardiovascular disease [5, 6].

Acute COPD exacerbations (AECOPD) are the main cause of morbidity in patients with COPD. While bacterial or viral infections are the most common causes [7], exacerbations can also be precipitated by other triggers [8]. Frequent exacerbations are associated with a faster decline in lung function, and poor clinical outcomes [8, 9]. *Haemophilus influenzae, Streptococcus pneumoniae,* and *Moraxella catarrhalis* are common bacterial infections [9] and rhinovirus is a common viral infection [3] linked to AECOPD.

Patients with COPD present with frequent respiratory infections that are often the cause for their AECOPD. Humoral immunity constitutes a major form of immune defense against respiratory infections. Studies have shown that approximately a quarter of patients with COPD have a defective humoral immunity and present with low immunoglobulin (Ig)G levels [10, 11]. In addition, among patients evaluated for lung transplantation, patients with COPD had lower IgG levels and increased likelihood for hypogammaglobulinemia compared to those with other lung diseases [12]. Moreover, IgG levels have been shown to correlate with the risk for exacerbations and hospitalizations in patients with COPD, an association seen even in patients with IgG levels in the normal range [10]. The likelihood of hypogammaglobulinemia increases with increased severity of COPD and is associated with increased mortality [8]. IgG subclass deficiency, especially IgG2 deficiency, is also common in patients with COPD [10, 11] and IgG subclass levels correlate with poor outcomes [10]. The cause for low immunoglobulin levels in patients with COPD is multifactorial including, possibly, the fact that oral corticosteroids may decrease IgG levels [11].

Little is known about the clinical significance of low IgG levels in patients with COPD. In two retrospective studies that evaluated the response of patients with COPD to IgG replacement therapy [13, 14], IgG replacement decreased one or more of the following outcomes: frequency of AECOPD, courses of oral corticosteroids, cumulative total dose of corticosteroids, use of antibiotics or hospitalization. However, no data exist from randomized placebo-controlled trials or other high-quality research studies.

In the study presented here, we hypothesized that decreased serum immunoglobulins levels in patients presenting with a severe COPD exacerbation are associated with worse outcomes. We tested this hypothesis in a cohort of patients with COPD recruited prospectively at hospitalization for an AECOPD. We measured immunoglobulin levels at admission and correlated these levels with length of stay during the index admission and the likelihood for repeated ED visits and/or hospitalization in the next year.

## Methods

### Patient population

**Fig. 1:**
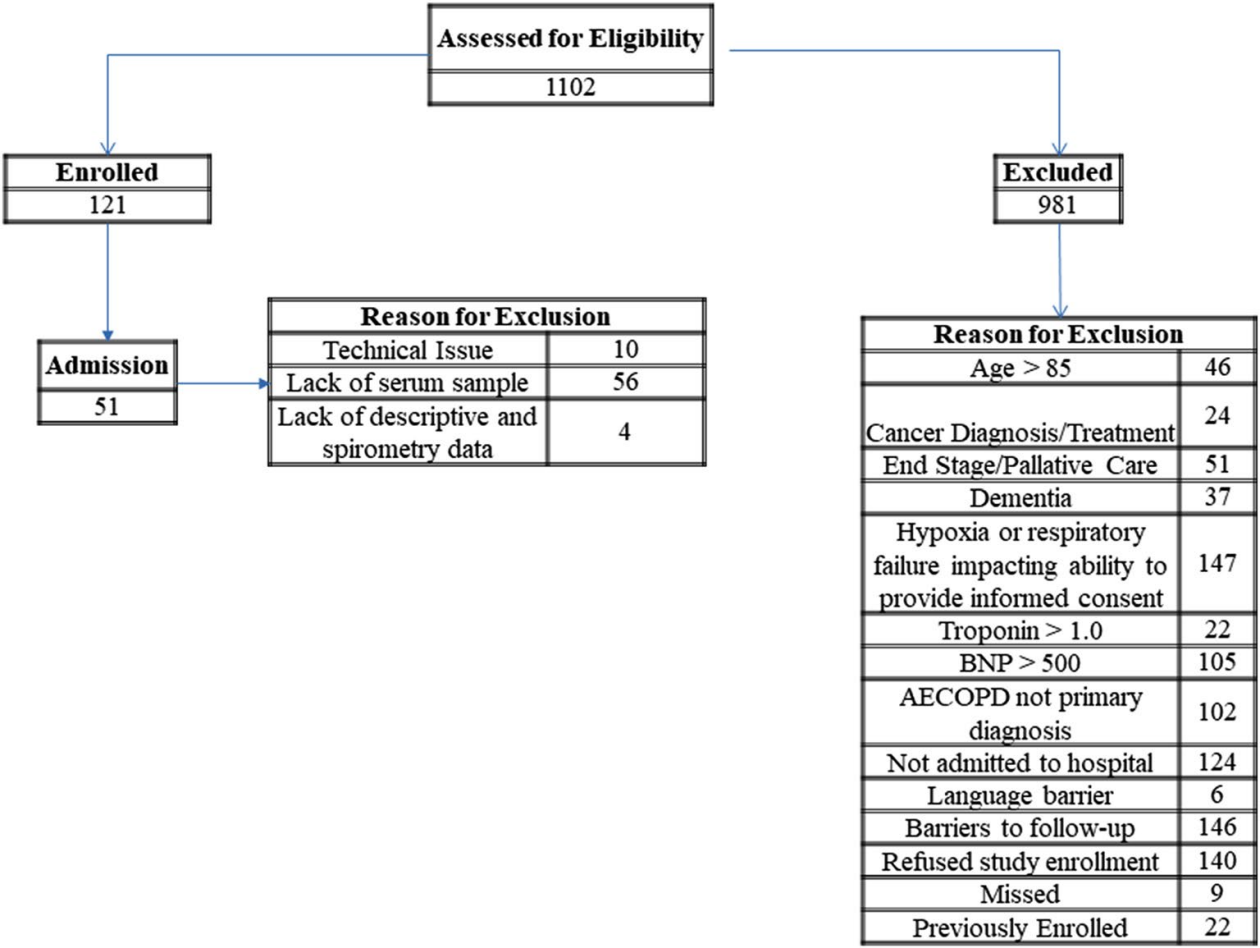
Flow diagram of sample size included in the study

The patients included in this study were originally recruited as a part of a larger prospective study to examine inflammation and cardiovascular risks in patients hospitalized for COPD at the University of Alberta Hospital (UAH), in Edmonton, Canada. The study was approved by the University of Alberta health research ethics review board (approval number Pro00038838). After informed consent, 55 patients were recruited on admission to the hospital for an acute COPD exacerbation from 6th February 2014 to July 31st 2017. From these subjects, 51 had a previous diagnosis of COPD by a respirologist and are included in the current study (Fig. 1). There was no study-specific therapeutic intervention during the index hospitalization. Patients were treated according to the standards and algorithms for AECOPD in use at the UAH.

Demographics were recorded at admission. The following clinical outcomes were evaluated for the 12 months after the index admission for COPD exacerbation: length of hospital stay at index admission (LOHSIA); emergency department visits (EDV); number of readmissions since hospital discharge date of index admission (readmission); AECOPD related hospital readmissions since hospital discharge date of index admission (AECOPD readmission).

### Immunoglobulin evaluation

Blood was collected prospectively < 24 h after admission in BD Vacutainer serum tubes. Blood was left to clot at room temperature (30 min) and then centrifuged at 1200 rpm (10 min). Serum was then separated, aliquoted, and stored at − 80ºC until use.

Immunoglobulin levels (IgG, IgA, and IgM) were measured in these serum samples by Alberta Precision Laboratories by turbid metric process. The normal ranges of immunoglobulins for the test used were IgG (6.94–16.18 g/L), IgA (0.70–4.00 g/L) and IgM (0.60–3.0 g/L).

### Statistical analysis

For immunoglobulin levels and other continuous clinical outcome, median and interquartile range (IQR) values are reported. Discrete variables are reported as percentages. Correlation between immunoglobulin (IgG, IgM, and/or IgA) levels with clinical outcomes and demographics were analyzed by Spearman’s correlation coefficient test in the whole population and separately in the subgroups with low and normal immunoglobulin values. Bivariate comparisons of clinical outcomes and demographics between subjects with low vs normal IgG or IgM were performed using Mann–Whitney U test for continuous variables and using the Chi-squared test for categorical variables.

To explore the factors associated with LOHSIA, univariate analysis as well as negative binomial regression were used. The regression analysis was performed using STATA Release 16 (StataCopr LP, College Station, Texas, USA). A p-value ≤ 0.05 was considered statistically significant.

## Results

The cohort presented in this study was recruited to examine a larger question around cardiovascular consequences of a COPD exacerbation. Recruited subjects were hospitalized for a COPD exacerbation, but detailed information regarding their previous medical history was not collected. All characteristics of the cohort presented in this manuscript were collected during the hospital admission and for some of these we do not have complete data.

Demographics and other data regarding the 51 patients included in our study are shown in Table 1. The median age of study patients was 67 years, and the median body mass index (BMI) 26.0 kg/m^2^. Fifty-three percent of subjects were female. Twenty-four percent of patients were current smokers with a median number of pack years of 45.0. The median FEV_1_ (% predicted) was 37.0% and the median FEV_1_/FVC ratio 42.5%. The median and IQR of immunoglobulin levels were IgG: 8.27 (6.53–10.07), IgA: 1.97 (1.49–2.69), IgM: 0.91 (0.49–1.37). Among the 51 patients, 35 (68.6%) had normal serum IgG levels, 14 (27.5%) had hypogammaglobulinemia and 2 (3.9%) subjects had IgG levels over the normal range. Forty-seven (92.2%) subjects had normal IgA, 1 (2.0%) had low IgA and 3 (5.9%) subjects had IgA levels over the normal range. Similarly, 34 (66.7%) subjects had normal serum IgM levels, 16 (31.4%) had low IgM and 1 (2.0%) subject had IgM levels over the normal range. In total 47.1% of the population had at least one immunoglobulin below the normal range. Among the 51 patients, 48 (82.8%) subjects had taken systemic corticosteroid and 46 (79.3%) subjects had taken antibiotics.

**Table 1:** Demographics and other characteristics of study subjects

Parameters	(N = 51)
Age (years) (median {IQR})	67 (61.0–75.0)
Sex—female (n {%})	26 (53%)
BMI* (kg/m2) (median {IQR})	26.3 (20.2–32.9)
Current smoking* (n {%})	12 (24.4%)
Number of pack years* (median {IQR})	45.0 (24.1–52.5)
FEV_1_** (% predicted) (median {IQR})	37.0 (22.5–49.9)
FEV_1_/FVC** (%). (median {IQR})	42.5 (36.6–50.4)
IgG (median {IQR})	8.27 (6.53–10.07)
IgA (median {IQR})	1.97 (1.49–2.69)
IgM (median {IQR})	0.91 (0.49–1.37)
Subjects taking systemic corticosteroid (%)	48 (82.8%)
Subjects taking antibiotics (%)	46 (79.3%)

*BMI* Body mass index, *FEV*_*1*_ Forced expiratory volume in 1 s, *FVC* Forced vital capacity, *Ig* immunoglobulin, *IQR* interquartile range, *y* year

^*^n = 50, **n = 29, because of missing data points

We then compared subjects with low IgG or IgM levels with those with normal IgG and IgM levels, respectively. Subjects with immunoglobulin levels over the normal limits were excluded from this analysis, as those were very small groups and the etiology of increased immunoglobulin levels was not clear. Since only one individual had low IgA levels, we did not perform similar analysis based on IgA.

Subjects having low IgG had significantly lower BMI compared to subjects having normal IgG (*p* = 0.009); however, there was no other differences in the demographic and lung function data between the two groups (Table 2). Subjects with low IgG levels also had lower IgA levels compared to those with normal IgG levels, but no difference in IgM levels (Table 2). There were no differences in any of the clinical outcomes between subjects with normal and those with low IgG levels (Table 2).

**Table 2:** Clinical outcome comparison between subjects with low vs normal IgG

	Low IgG (N = 14)	Normal IgG (N = 35)	*p*
Demographics			
Age years (median{IQR})	73 (65–79)	66 (58–73)	0.132
Sex—female (n {%})	7 (50%)	19 (54.3%)	1.000
BMI* (kg/m2) (median {IQR})	22.2 (19.8–24.0)	29.2/34 (21.8–34.2)	**0.009**
FEV_1_** (% predicted) (median. {IQR})	37.5 (23.6–66.4)	37.4 (22.4–50.0)	0.799
FEV_1_/FVC** (%) (median {IQR})	39.8 (36.9–52.9)	43.0 (35.1–48.5)	0.879
Current Smoking* (n {%})	4 (30.8%)	8 (22.9%)	0.710
Number of pack years***(median {IQR})	50.0 (24.8–57.5)	45.0 (21.0–50.0)	0.500
Subjects taking systemic corticosteroid (%)	13 (92.9%)	33 (94.3%)	1.000
Subjects taking antibiotics (%)	13 (92.9%)	31 (88.6%)	1.000
Immunoglobulin levels			
IgG (median {IQR})	6.00 (5.3–6.1)	9.34 (7.6–10.5)	** < 0.0001**
IgA (median {IQR})	1.46 (0.9–1.8)	2.32 (1.7–2.9)	** < 0.0001**
IgM (median {IQR})	0.69 (0.4–1.1)	0.91 (0.5–1.4)	0.192
Clinical outcomes			
Length of hospital stay (median {IQR})	2.5 (2.0–5.3)	4.0 (3.0–6.0)	0.245
Emergency department visits (median {IQR})	2.0 (0.7–4.7)	1.0 (0.0–4.0)	0.297
Readmissions (median {IQR})	1.5 (0.0–3.0)	0.0 (0.0–2.0)	0.320
AECOPD related readmissions (median {IQR})	1.0 (0.0–2.0)	0.0 (0.0–1.0)	0.195

Bold values denote statistical significance at the p < 0.05 level

*AECOPD* Acute chronic obstructive pulmonary disease exacerbation, *BMI* body mass index, *FEV*_*1*_ forced expiratory volume in 1 s, *FVC* forced vital capacity, *Ig* immunoglobulin, *y* year

^*^n = 14 vs 34 for Low IgG vs Normal IgG

^**^n = 8 vs 20 for Low IgG vs Normal IgG

^***^n = 13 vs 35 for Low IgG vs Normal IgG because of missing data points

There was no significant difference in demographics and lung function data between subjects with normal and low IgM levels. Patients with low IgM levels had longer hospital stay during the index admission compared to patients with normal IgM levels (6.0 vs. 3.0 days, *p* = 0.003) (Table 3). This difference remained significant following the multi-variable regression analysis (IRR: 1.79; 95% CI: 1.16–2.75) for patients with low IgM. vs. normal IgM levels. (Table 4). While age and biological sex were not significant in the univariate analysis, these variables were considered clinically relevant and therefore retained as model factors to control their potential confounding effects. There were no differences in any of the other clinical outcomes studied.

**Table 3:** Clinical outcome comparison between subjects with low vs normal IgM

	Low IgM (N = 16)	Normal IgM (N = 34)	*p*
Demographics			
Age years (median {IQR})	70.5 (65.5–73.0)	65 (58–79)	0.235
Sex—female (n {%})	7(43.8%)	20(58.8%)	0.373
BMI* (kg/m2) (median {IQR})	25.6 (22.6–33.5)	26.4 (19.4–31.9)	0.456
FEV_1_** (% predicted) (median. {IQR})	43.9 (21.6–68.1)	35.1 (24.5–44.4)	0.270
FEV_1_/FVC** (%) (median {IQR})	46.7 (38.8–52.2)	41.1 (32.7–52.3)	0.292
Current Smoking* (n {%})	6/16 (37.5%)	6/33 (18.2%)	0.169
Number of pack years***(median {IQR})	50.0 (35.1–58.5)	40.0 (20.2–50.0)	0.069
Subjects taking systemic corticosteroid (%)	15 (93.8%)	32 (94.1%)	1.000
Subjects taking antibiotics (%)	15 (93.8%)	30 (88.2%)	1.000
Immunoglobulin			
IgG (median {IQR})	8.15 (6.00–10.63)	8.15 (6.99–10.06)	0.519
IgA (median {IQR})	1.84 (1.32–3.40)	2.04 (1.53–2.65)	0.685
IgM (median {IQR})	0.41 (0.37–0.48)	1.10 (0.85–1.44)	** < 0.0001**
Clinical outcome			
Length of hospital stay (median {IQR})	6.00 (4.00–8.75)	3.00 (2.00–5.00)	**0.003**
Emergency department visit number (median {IQR})	1.00 (0.00–3.75)	1.50 (0.00–4.00)	0.633
Readmission (median {IQR})	0.50 (0.00–2.00)	1.00 (0.00–3.00)	0.597
AECOPD related hospital readmission (median {IQR})	0.50 (0.00–1.75)	0.00 (0.00–2.00)	0.927

Bold values denote statistical significance at the p < 0.05 level

*AECOPD* Acute chronic obstructive pulmonary disease exacerbation, *BMI* body mass index, *FEV*_*1*_ forced expiratory volume in 1s, *FVC* forced vital capacity, *Ig* immunoglobulin, *y* year

^*^n = 16 vs 33 for Low IgM vs Normal IgM

^**^n = 10 vs 18 for Low IgM vs Normal IgM because of missing data points

**Table 4:** Factors associated with length of hospital stay among patients hospitalized for an acute COPD exacerbation

Variable	Adjusted IRR (95% CI)	*p*
Age	1.00 (0.98–1.02)	0.888
Sex Male Female	Reference 0.82 (0.54–1.23)	0.338
IgM Normal Low	Reference 1.79 (1.16–2.75)	**0.008**

Bold value denotes statistical significance at the p < 0.05 level

*IRR* Incidence rate ratio compared to the reference category the ratio of expected length of hospital stay

To understand whether immunoglobulin levels as continuous variables correlated with any clinical outcome, we performed correlation analysis of immunoglobulin levels and clinical outcomes for the whole population but also separately for the subgroups with low or normal immunoglobulin levels (Table 5). We excluded subjects with increased immunoglobulin levels from this analysis, as we also did above. Since there was only 1 subject with low IgA, we did not do this analysis for subjects with low IgA levels. In the low IgG group, there was a trend for negative correlation between serum IgG levels and AECOPD-related hospital admissions (R = − 0.527, p = 0.051), while no such correlation existed in the patients with normal IgG (Table 5). There was no correlation between IgM levels and clinical outcomes in any of the two IgM groups but in overall group, there was negative correlation between serum IgM levels and length of hospital stay (R = − 0.317, p = 0.024).

**Table 5:** Correlation of IgG, IgM and IgA levels with clinical outcomes

Clinical Outcomes	IgG			IgM			IgA
	All (N = 51)	Low IgG (N = 14)	Normal IgG (N = 35)	All (N = 51)	Low IgM (N = 16)	Normal IgM (N = 34)	All (N = 51)
Length of hospital stay	R = 0.275 *p* = 0.051	R = − 0.439 *p* = 0.116	R = 0.262 *p* = 0.128	R = − 0.317 *p* = **0.024**	R = 0.249 *p* = 0.353	R = 0.046 *p* = 0.798	R = 0.198 *p* = 0.163
Emergency department visits	R = − 0.052 *p* = 0.717	R = − 0.267 *p* = 0.357	R = 0.095 *p* = 0.586	R = − 0.110 *p* = 0.441	R = 0.012 *p* = 0.965	R = − 0.260 *p* = 0.138	R = − 0.232 *p* = 0.102
Readmissions	R = − 0.005 *p* = 0.973	R = − 0.469 *p* = 0.090	R = 0.174 *p* = 0.318	R = 0.014 *p* = 0.920	R = 0.086 *p* = 0.752	R = − 0.042 *p* = 0.812	R = − 0.070 *p* = 0.624
AECOPD related hospital readmissions	R = − 0.104 *p* = 0.467	R = − 0.527 *p* = 0**.**053	R = − 0.019 *p* = 0.914	R = − 0.033 *p* = 0.816	R = 0.250 *p* = 0.351	R = − 0.073 *p* = 0.680	R = − 0.116 *p* = 0.419

Bold value denotes statistical significance at the p < 0.05 level

*AECOPD* Acute chronic obstructive pulmonary disease exacerbation, *R* Correlation coefficient

N = 2, IgG > 16.18 g/L or higher than normal range

N = 1, IgM > 3.0, or higher than normal range

## Discussion

In this study, we analyzed immunoglobulin levels in a cohort of patients with severe COPD who were admitted to hospital for an AECOPD and found that low IgG and IgM levels were common among these patients. Moreover, IgM levels were associated with a longer hospital stay during the index admission for COPD exacerbation, even after adjustment for other variables. In addition, IgM levels in the whole sample showed a negative correlation with hospital stay during the index admission supporting the observation above. Conversely, IgG levels were not associated with any of the clinical outcomes of the study.

Forty-seven percentage of our cohort had below the normal range levels for at least one immunoglobulin class (27.5% had low IgG, 31.4% had low IgM and 2% had low IgA, while few subjects had more than one class low) at presentation. Unfortunately, we do not have longitudinal data on the cohort, so we do not know if this decrease represents an acute drop preceding the exacerbation, or a stable characteristic of these subjects with COPD. Previous studies have shown variable rates of IgG decrease in patients with COPD ranging from 11 to 20% [8]. Low IgG levels were associated with lower BMI and this association suggests that the nutritional status of the patient may be linked to their IgG levels. The exact reasons, however, for low immunoglobulin levels in patients with COPD are not clear. Many factors may be at play in addition to poor nutrition, including chronic disease or chronic oral corticosteroid use. A previous study has shown that patients with COPD on oral corticosteroids have lower IgG levels than those who were not on corticosteroids [11]; whether this association depends on the severity of the disease, or is linked directly to corticosteroid use, is not clear. It is unfortunate that we do not have any data on the severity and treatment of COPD prior to the index admission, or an evaluation of immunoglobulin levels prior to the index admission. This deficiency limits our ability to reach conclusions regarding the cause of decreased immunoglobulin levels in some members of our cohort.

Subjects with low IgM levels at presentation had higher length of stay for the index admission compared to those with normal IgM levels. This finding was further confirmed adjusting with age and biological sex. There are multiple potential explanations for this observation. Since the most frequent reason for COPD exacerbations is infections, low IgM may mean that the immune system is not able to mount an immediate response against the infectious agent and therefore these patients present with increased severity of infection that would require longer hospital stay. It would be beneficial to design a longitudinal study to understand whether there is a specific phenotype of patients presenting with AECOPD who have low IgM levels and whether this translates into an endotype with pathophysiological significance. If this were the case, this may be a population where focused efforts, such as close medical follow up, optimized medical treatment including prophylactic antibiotics, appropriate vaccinations and other preventive measures are needed to prevent subsequent infections. It would also have been interesting to have data on the bacteriology of infections leading to AECOPD, but we do not have such data in our study.

When the sample was subclassified into those with low and with normal IgG levels, we found a trend towards a negative correlation between the number of AECOPD-related hospital readmissions and serum IgG only in the subjects with decreased IgG levels. One limitation regarding this observation is that we had a very small number of patients with low IgG in the cohort we presented here. This observation may indicate that the degree of IgG level decrease is associated with an increased risk of infections. This observation is concordant with observations from a previous study that showed worse outcomes with decreasing IgG levels, although in that case the difference could be seen across the whole spectrum of IgG levels [10]. Larger prospective studies in similar populations will provide more robust evidence of the associations between IgG levels and the course of COPD.

The study has also several limitations. First, the population is small, and the observation needs to be validated in a larger study of patients presenting with COPD exacerbations. Second, the measurement of immunoglobulins in our study was performed in serum collected during the presentation with AECOPD. As mentioned also above, we have no data on severity of the disease or use or oral corticosteroid use prior to the index admission. Therefore, it cannot be determined whether immunoglobulin levels change acutely around the time of an AECOPD and whether immunoglobulin levels during convalescent periods would also correlate with the same outcomes. Future prospective studies with immunoglobulin level evaluation in stable patients with COPD of various degree of severity and evaluation for the cause of decreased levels, when present, will be required to improve our understanding of the effects of immune dysfunction in the course of COPD. Third, there was limited granular data on the exacerbation, management, and previous health, which all impact the primary outcomes of length of stay. Fourth, there is no data demonstrating acute alterations of immunoglobulin levels during an exacerbation. This may be a possibility due to the likely presence of a concurrent infection and the increased dose of mediations, especially oral steroids, subjects may be taking during exacerbations. Finally, the study was conducted in Canada, where access to health care and drugs is federally regulated through the Canada Health Act. The study should be reproduced in other settings.

## Conclusion

In patients presenting with a COPD exacerbation requiring hospitalization, low IgM levels are associated with longer length of hospital stay. This category of patients may represent a specific patient phenotype that requires alternative management approaches to prevent subsequent infections. In this sample, low IgG levels did not correlate with the primary clinical outcomes. Larger prospective studies are required to fully understand the biological significance of low immunoglobulin levels in patients with COPD.

## Data Availability

The datasets used and/or analysed during the current study are available from the corresponding author on reasonable request.
